# Crystal structure of *trans*-*N*,*N*′-bis­(3,5-di-*tert*-butyl-2-hy­droxy­phen­yl)oxamide methanol monosolvate

**DOI:** 10.1107/S205698901600880X

**Published:** 2016-06-10

**Authors:** Miguel-Ángel Velázquez-Carmona, Sylvain Bernès, Francisco Javier Ríos-Merino, Yasmi Reyes Ortega

**Affiliations:** a Centro de Química, Instituto de Ciencias, Benemérita Universidad Autónoma de Puebla, 72570 Puebla, Pue., Mexico; b Instituto de Física, Benemérita Universidad Autónoma de Puebla, Av. San Claudio y 18 Sur, 72570 Puebla, Pue., Mexico

**Keywords:** crystal structure, oxamide, disorder, solvate, hydrogen bonding

## Abstract

The here crystallized oxamide was previously characterized as an unsolvated species [Jímenez-Pérez *et al.* (2000 ▸). *J. Organomet. Chem*. **614–615**, 283–293], and is now reported with methanol as a solvent of crystallization, C_30_H_44_N_2_O_4_·CH_3_OH, in a different space group. The introduction of the solvent influences neither the mol­ecular symmetry of the oxamide, which remains centrosymmetric, nor the mol­ecular conformation. However, the unsolvated mol­ecule crystallized as an ordered system, while many parts of the solvated crystal are disordered. The hy­droxy group in the oxamide is disordered over two chemically equivalent positions, with occupancies 0.696 (4):0.304 (4); one *tert*-butyl group is disordered by rotation about the C—C bond, and was modelled with three sites for each methyl group, each one with occupancy 1/3. Finally, the methanol solvent, which lies on a twofold axis, is disordered by symmetry. The disorder affecting hy­droxy groups and the solvent of crystallization allows the formation of numerous supra­molecular motifs using four hydrogen bonds, with N—H and O—H groups as donors and the oxamide and methanol mol­ecule as acceptors.

## Abstract 2

In the title solvate, the oxamide derivative presents the same conformation as in the unsolvated mol­ecule, but the lattice solvent introduces disorder for various functional groups.

## Chemical context   

1,2-Bis-(3,5-di-*tert*-butyl-2-hy­droxy­phen­yl)oxamide has been synthesized by two different routes, reported in the literature (Jímenez-Pérez *et al.*, 2000 ▸; Beckmann *et al.*, 2003 ▸). For several oxamide derivatives, NMR and crystallographic studies have shown that these compounds have the same conformation in the solid state and in solution: a planar structure stabilized by an intra­molecular three-centre hydrogen bond forming two five-membered rings (Martínez-Martínez *et al.*, 1993 ▸, 1998 ▸). Other studies of the polymerization of ethyl­ene showed that Zr complexes bearing oxamide ligands are active as catalyst (Güizado-Rodríguez *et al.*, 2007 ▸). Phenyl­oxamides have also been reported as light stabilizers for plastics (Burdet *et al.*, 1972 ▸).
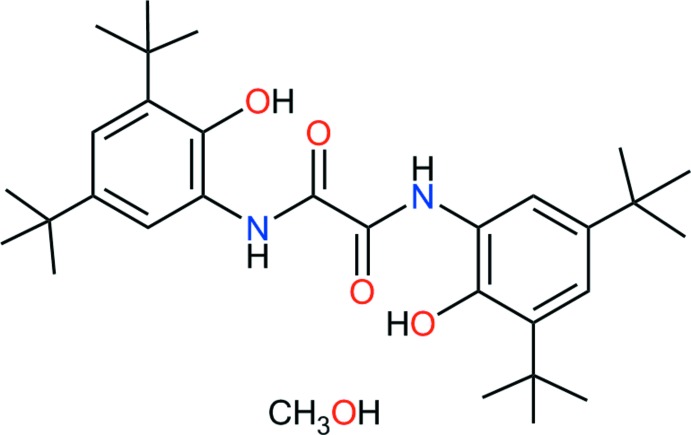


While attempting to coordinate 1,2-bis-(3,5-di-*tert*-butyl-2-hy­droxy­phen­yl)oxamide to first-row transition metals in methanol, we obtained crystals of the title solvate, for which we report here the mol­ecular and crystal structures.

## Structural commentary   

**Figure 1:**
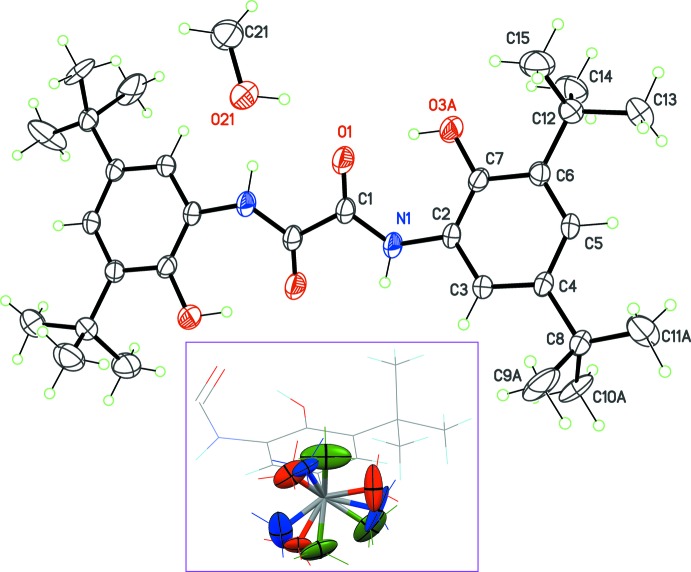
The structure of the title solvate, with atom labelling and displacement ellipsoids drawn at the 30% probability level. A single site for the disordered groups is shown, and non labelled atoms are generated by inversion symmetry. The inset represents the resolved disorder in the *tert*-butyl group C8/C9/C10/C11 (colour code: red, green and blue ellipsoids are for sites *A*, *B* and *C*, respectively).

The *trans*-oxamide derivative lies on an inversion centre, placed at the midpoint of the central C1—C1^i^ bond [symmetry code: (i) 1 − *x*, −*y*, 1 − *z*], and the methanol mol­ecule is placed close to the twofold axis of the *C*2/*c* space group, and was then refined with its occupancy constrained to 1/2 (Fig. 1 ▸). The dimensions for the oxamide mol­ecule are very similar to those reported for the unsolvated crystal (Jímenez-Pérez *et al.*, 2000 ▸).

**Figure 2:**
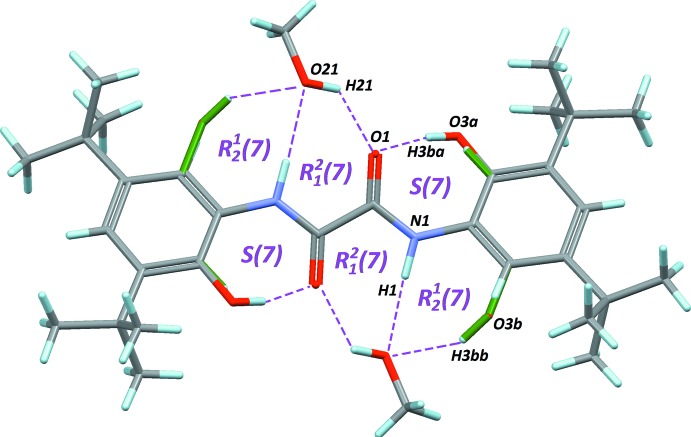
*S* and *R* motifs formed *via* hydrogen bonding in the title solvate. Disordered sites O3*A* and O3*B* are retained, since they participate in different patterns. All hydrogen bonds listed in Table 1 ▸ are represented by dashed lines.

The mol­ecular conformation is not planar, and can be described using the dihedral angle between the oxamide core C1/O1/N1 and the benzene ring C2–C7. In the title solvate, this angle is 32.4 (2)°, slightly smaller than the same angle observed in the unsolvated crystal, 38.4°. A comparison of conformations stabilized for this mol­ecule shows that a planar conformer is obtained only if amine and hy­droxy groups are deprotonated to form a tetra­anion, which is then able to coordinate a metal centre (*e.g*. Beckmann *et al.*, 2003 ▸). The twisted conformation for the neutral mol­ecule is probably a consequence of the formation of an intra­molecular hydrogen bond between hy­droxy and carbonyl groups (Table 1 ▸, entry 1). The resulting motif is an *S*(7) self-associating pattern having an envelope shape, in order to bring the O—H⋯O angle as close as possible to 180°. The involved OH group is disordered over two chemically equivalent positions on the benzene ring, C7 and C3. However, the most populated site, O3*A*, which has a site occupancy factor of 0.696 (4), is that forming this contact (Fig. 2 ▸). Because of the centrosymmetric character of the mol­ecule, two occurrences of the *S*(7) motif are stabilizing the twisted conformation.

Other potential intra­molecular hydrogen bonds starting from the amine groups N1 are present in the mol­ecule, forming other *S* rings with lower degree. However, these contacts, N1—H1⋯O1 and N1—H1⋯O3*B*, are not relevant for the mol­ecular conformation, since their *D*—H⋯*A* angles are close to 100°, corresponding to a stabilization energy close to 0 kJ mol^−1^ (Wood *et al.*, 2009 ▸).

## Supra­molecular features   

The introduction of methanol changes the original *P* 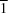 crystal symmetry (Jímenez-Pérez *et al.*, 2000 ▸) to *C*2/*c* (Table 2 ▸). The methanol molecule is located in close proximity to the oxamide, and behaves both as a donor and acceptor for hydrogen bonding (Table 1 ▸, entries 2–4). Discrete O—H⋯O(methanol) weak bonds are formed with the disordered hy­droxy group O3*B* of the oxamide, as well as N—H⋯O(methanol) with the amine groups. As a result, 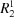
(7) rings are formed (Fig. 2 ▸). The last heteroatom involved in hydrogen bonding is the carbonyl O atom O1, acting as an acceptor (Table 1 ▸, entry 4), to form 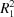
(7) rings.

## Database survey   

The oxamidate derived from the title oxamide has been used extensively for coordination chemistry. It is possible to find one report in the literature for zinc clusters with 1,2-bis-(3,5-di-*tert*-butyl-2-hy­droxy­phen­yl)oxamidate (Rufino-Felipe *et al.*, 2016 ▸). In these complexes, the crystal structures exhibit an octa­nuclear Zn_8_ cage and a hexa­nuclear Zn_6_ cage, where the nuclearity of the cages is driven by the solvent. Other compounds with Si or Ge (Jiménez-Pérez *et al.*, 2007 ▸) are described as bimetallic hexa­cyclic symmetric heterocycles, with hypervalent Si and Ge centres. For Sn compounds (Jímenez-Pérez *et al.*, 2000 ▸; Contreras *et al.*, 2000 ▸), two penta- or hexa-coordinated Sn atoms are arranged in a hexa­cyclic symmetric planar array. For Fe and Ga complexes (Beckmann *et al.*, 2003 ▸; Bill *et al.*, 2002 ▸), the metal ions Ga^3+^ and Fe^3+^ are five-coordinate, with a distorted trigonal–bipyramidal geometry in a hexa­cyclic symmetric planar array. Finally, in Ti, Zr and Hf complexes (Güizado-Rodríguez *et al.*, 2007 ▸), the metal displays a planar structure similar to that observed in Sn complexes, but no X-ray structures were determined.

On the other hand, several phenol-oxamides have shown different conformations, ranging from completely flat (Weiss *et al.*, 2015 ▸) to arrangements where the oxamide group presents a tilt angle, or is even almost completely perpendic­ular to the plane of the aromatic rings (Wen *et al.*, 2006 ▸; Piotrkowska *et al.*, 2007 ▸). Piotrkowska’s group made a good analysis of the phenyl-oxamides and explained how the substituent groups on the aromatic rings and the presence of solvent influence the conformation of the oxamide group: hydrogen bonds and π–π stacking between aromatic rings are the main forces responsible for the assembly of mol­ecules within the crystal lattice. Thus, the steric effects of the bulky *o*-substituents cause twisting of the aryl ring from the oxamide plane, and inter­fere with the formation of hydrogen bonds (Piotrkowska *et al.*, 2007 ▸).

## Synthesis and crystallization   

The reaction of 100 mg (0.171 mmol) of disodium bis­(4,6-di-*tert*-butyl-1-oxo-phen­yl)oxamido and 81 mg (0.342 mmol) of NiCl_2_·6H_2_O in methanol with a molar ratio 1:2 afforded a dark-brown solution. An amount of maleic acid (79 mg, 0.684 mmol), intended as a bridging ligand, was then added, changing the colour of the solution to light green. After a few minutes under stirring, a cottony precipitate formed. The solution was filtered and the filtrate allowed to crystallize by solvent evaporation, affording needle-shaped green crystals. The green colour is due to a thin layer of nickel chloride covering the crystals. Some of these crystals were washed with methanol, giving colourless crystals (m.p. 496-497 K), used for X-ray crystallography.

Spectroscopic data: FT–IR (KBr, cm^−1^): 3501, 3355, 3274 (OH, NH), 1651 (C=O). ^1^H NMR (500 MHz, CDCl_3_) δ, p.p.m.: 9.54 (*s*, 2H, O***H***), 7.57 (*s*, 2H, N***H***), 7.32 (*d*, 2H, *J* = 2.3 Hz, Ph), 7.15 (*d*, 2H, *J* = 2.3 Hz, Ph), 3.51 (*s*, C***H***_3_OH), 1.61 (*s*, CH_3_O***H***), 1.48 (*s*, 18H, C***H***_3_C, ^*t*^Bu), 1.33 (*s*, 18H, C***H***_3_C, ^*t*^Bu). ^13^C NMR (100 MHz, CDCl_3_) δ, p.p.m.: 157.28 (***C***=O, oxamide), 145.82 (***C***—O, phenol), 143.31 (***C***, quaternary Ph), 139.77 (***C***, quaternary Ph), 123.99 (***C—***N), 123.17 (***C***H, Ph), 117.71 (***C***H, Ph), 35.40 (CH_3_ ***C***, ^*t*^Bu), 34.41 (CH_3_ ***C***, ^*t*^Bu), 31.45 (***C***H_3_C, ^*t*^Bu), 30.97 (***C***H_3_OH), 29.83 (***C***H_3_C, ^*t*^Bu).

## Refinement   

Crystal data, data collection and structure refinement details are summarized in Table 2 ▸. In the asymmetric unit, one *tert*-butyl group is severely disordered by rotation, and each methyl group was split over three sites, labelled *A*, *B* and *C*, with occupancy fixed to 1/3 (Fig. 1 ▸, inset). ADPs for these C atoms were restrained to approximate isotropic behaviour with a standard uncertainty of 0.1 Å^2^, and the nine atoms were restrained to have the same displacement parameters within 0.04 Å^2^ deviation. Finally, C8—methyl bond lengths were restrained to be equal with a standard uncertainty 0.02 Å. The hy­droxy group in the oxamide is disordered over two chemically equivalent sites, O3*A* and O3*B*, and their occupancies converged to 0.696 (4) and 0.304 (4), respectively. Finally, the methanol mol­ecule is disordered by symmetry over a twofold axis, and its occupancy was fixed at 1/2. The geometry for this mol­ecule was restrained with bond length C21—O21 = 1.45 (1) Å. All H atoms bonded to C atoms were placed in idealized positions and refined as riding atoms, with C—H bond lengths fixed to 0.93 (aromatic) or 0.96 Å (methyl groups) and *U*_iso_(H) = *xU*_eq_(C) with *x* = 1.2 (aromatic) or 1.5 (methyl groups). H atoms bonded to heteroatoms were found in difference maps and refined with restraints applied to the O—H bond lengths: 0.90 (1) Å (methanol) and 0.85 (2) Å (hy­droxy). For these H atoms *U*_iso_(H) = *xU*_eq_(carrier atom), with *x* = 1.2 (NH) or *x* = 1.5 (OH).

## Supplementary Material

Crystal structure: contains datablock(s) I, global. DOI: 10.1107/S205698901600880X/su5302sup1.cif

Structure factors: contains datablock(s) I. DOI: 10.1107/S205698901600880X/su5302Isup2.hkl

Supporting information file. DOI: 10.1107/S205698901600880X/su5302Isup3.cml

CCDC reference: 1482681

Additional supporting information: crystallographic information; 3D view; checkCIF report

## Figures and Tables

**Table 1:** Hydrogen-bond geometry (Å, °)

*D*—H⋯*A*	*D*—H	H⋯*A*	*D*⋯*A*	*D*—H⋯*A*
O3*A*—H3*BA*⋯O1	0.88 (2)	1.71 (2)	2.578 (2)	167 (4)
O3*B*—H3*BB*⋯O21^i^	0.85 (2)	2.14 (2)	2.612 (9)	114 (4)
N1—H1⋯O21^ii^	0.95 (2)	2.27 (2)	3.140 (6)	152 (2)
O21—H21⋯O1	0.90 (1)	2.01 (3)	2.744 (6)	138 (4)

Symmetry codes: (i) 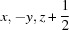; (ii) 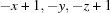.

**Table 2:** Experimental details

Crystal data	
Chemical formula	C_30_H_44_N_2_O_4_·CH_4_O
*M*_r_	528.71
Crystal system, space group	Monoclinic, *C*2/*c*
Temperature (K)	298
*a*, *b*, *c* (Å)	27.614 (3), 10.5561 (11), 10.6875 (9)
β (°)	91.722 (9)
*V* (Å^3^)	3113.9 (5)
*Z*	4
Radiation type	Mo *K*α
μ (mm^−1^)	0.08
Crystal size (mm)	0.62 × 0.10 × 0.07

Data collection	
Diffractometer	Agilent Xcalibur Atlas Gemini
Absorption correction	Multi-scan (*CrysAlis PRO*; Agilent, 2013 ▸)
*T*_min_, *T*_max_	0.664, 1.000
No. of measured, independent and observed [*I* > 2σ(*I*)] reflections	16488, 3188, 2152
*R*_int_	0.038
(sin θ/λ)_max_ (Å^−1^)	0.625

Refinement	
*R*[*F* ^2^ > 2σ(*F* ^2^)], *wR*(*F* ^2^), *S*	0.053, 0.155, 1.01
No. of reflections	3188
No. of parameters	263
No. of restraints	174
H-atom treatment	H-atom parameters constrained
Δρ_max_, Δρ_min_ (e Å^−3^)	0.14, −0.15

Computer programs: *CrysAlis PRO* (Agilent, 2013 ▸), *SHELXS2013* and *SHELXTL* (Sheldrick, 2008 ▸), *SHELXL2014* (Sheldrick, 2015 ▸), *Mercury* (Macrae *et al.*, 2008 ▸) and *CifTab* (Sheldrick, 2015 ▸).

## supplementary crystallographic information

### Crystal data

**Table:**

C_30_H_44_N_2_O_4_·CH_4_O	*D*_x_ = 1.128 Mg m^−^^3^
*M**_r_* = 528.71	Melting point: 496 K
Monoclinic, *C*2/*c*	Mo *K*α radiation, λ = 0.71073 Å
*a* = 27.614 (3) Å	Cell parameters from 3123 reflections
*b* = 10.5561 (11) Å	θ = 4.3–28.5°
*c* = 10.6875 (9) Å	µ = 0.08 mm^−^^1^
β = 91.722 (9)°	*T* = 298 K
*V* = 3113.9 (5) Å^3^	Needle, colourless
*Z* = 4	0.62 × 0.10 × 0.07 mm
*F*(000) = 1152	

### Data collection

**Table:**

Agilent Xcalibur Atlas Gemini diffractometer	3188 independent reflections
Radiation source: Enhance (Mo) X-ray Source	2152 reflections with *I* > 2σ(*I*)
Graphite monochromator	*R*_int_ = 0.038
Detector resolution: 10.5564 pixels mm^-1^	θ_max_ = 26.4°, θ_min_ = 2.9°
ω scans	*h* = −34→34
Absorption correction: multi-scan (CrysAlis PRO; Agilent, 2013)	*k* = −13→13
*T*_min_ = 0.664, *T*_max_ = 1.000	*l* = −13→13
16488 measured reflections	

### Refinement

**Table:**

Refinement on *F*^2^	Primary atom site location: structure-invariant direct methods
Least-squares matrix: full	Secondary atom site location: difference Fourier map
*R*[*F*^2^ > 2σ(*F*^2^)] = 0.053	Hydrogen site location: mixed
*wR*(*F*^2^) = 0.155	H-atom parameters constrained
*S* = 1.01	*w* = 1/[σ^2^(*F*_o_^2^) + (0.0878*P*)^2^ + 2.0368*P*] where *P* = (*F*_o_^2^ + 2*F*_c_^2^)/3
3188 reflections	(Δ/σ)_max_ < 0.001
263 parameters	Δρ_max_ = 0.14 e Å^−^^3^
174 restraints	Δρ_min_ = −0.15 e Å^−^^3^
0 constraints	

### Fractional atomic coordinates and isotropic or equivalent isotropic displacement parameters (Å^2^)

**Table:**

	*x*	*y*	*z*	*U*_iso_*/*U*_eq_	Occ. (<1)
O1	0.47428 (5)	0.15121 (14)	0.49515 (12)	0.0698 (4)	
N1	0.46117 (5)	−0.01249 (16)	0.62855 (13)	0.0512 (4)	
H1	0.4704 (7)	−0.099 (2)	0.6394 (17)	0.061*	
C1	0.48192 (6)	0.04271 (18)	0.53215 (15)	0.0483 (4)	
C2	0.42414 (6)	0.03520 (16)	0.70695 (14)	0.0431 (4)	
C3	0.39184 (6)	−0.05361 (16)	0.75100 (15)	0.0462 (4)	
H3A	0.3947	−0.1378	0.7268	0.055*	0.696 (4)
O3A	0.45301 (8)	0.2536 (2)	0.70552 (18)	0.0704 (8)	0.696 (4)
H3BA	0.4609 (13)	0.230 (4)	0.630 (2)	0.106*	0.696 (4)
O3B	0.39663 (18)	−0.1851 (4)	0.7200 (5)	0.0751 (18)	0.304 (4)
H3BB	0.413 (3)	−0.248 (6)	0.693 (8)	0.113*	0.304 (4)
C4	0.35521 (6)	−0.01909 (16)	0.83061 (14)	0.0449 (4)	
C5	0.35307 (6)	0.10772 (16)	0.86394 (14)	0.0463 (4)	
H5A	0.3289	0.1325	0.9176	0.056*	
C6	0.38452 (6)	0.19979 (16)	0.82263 (14)	0.0447 (4)	
C7	0.42103 (6)	0.16127 (17)	0.74191 (14)	0.0463 (4)	
H7A	0.4430	0.2201	0.7122	0.056*	0.304 (4)
C8	0.31825 (7)	−0.11625 (18)	0.87695 (16)	0.0560 (5)	
C9A	0.2998 (9)	−0.196 (2)	0.7701 (16)	0.112 (9)	0.3333
H9AA	0.2768	−0.2562	0.7999	0.168*	0.3333
H9AB	0.3263	−0.2398	0.7338	0.168*	0.3333
H9AC	0.2843	−0.1426	0.7081	0.168*	0.3333
C10A	0.3456 (6)	−0.2269 (12)	0.9387 (13)	0.071 (5)	0.3333
H10A	0.3228	−0.2880	0.9679	0.106*	0.3333
H10B	0.3651	−0.1962	1.0082	0.106*	0.3333
H10C	0.3661	−0.2660	0.8788	0.106*	0.3333
C11A	0.2718 (5)	−0.0582 (13)	0.926 (2)	0.147 (8)	0.3333
H11A	0.2506	−0.1245	0.9524	0.220*	0.3333
H11B	0.2559	−0.0096	0.8610	0.220*	0.3333
H11C	0.2796	−0.0040	0.9960	0.220*	0.3333
C9B	0.2772 (7)	−0.113 (2)	0.7849 (19)	0.179 (13)	0.3333
H9BA	0.2826	−0.0473	0.7245	0.268*	0.3333
H9BB	0.2477	−0.0957	0.8274	0.268*	0.3333
H9BC	0.2746	−0.1931	0.7430	0.268*	0.3333
C10B	0.3368 (7)	−0.1974 (19)	0.9810 (14)	0.124 (8)	0.3333
H10D	0.3702	−0.1773	0.9994	0.185*	0.3333
H10E	0.3341	−0.2849	0.9571	0.185*	0.3333
H10F	0.3181	−0.1827	1.0540	0.185*	0.3333
C11B	0.3052 (9)	−0.0774 (19)	1.0082 (13)	0.128 (7)	0.3333
H11D	0.2875	0.0008	1.0049	0.192*	0.3333
H11E	0.3343	−0.0664	1.0585	0.192*	0.3333
H11F	0.2856	−0.1421	1.0444	0.192*	0.3333
C9C	0.2858 (7)	−0.170 (2)	0.7759 (11)	0.078 (5)	0.3333
H9CA	0.3051	−0.2076	0.7124	0.117*	0.3333
H9CB	0.2662	−0.1040	0.7395	0.117*	0.3333
H9CC	0.2653	−0.2338	0.8107	0.117*	0.3333
C10C	0.3437 (7)	−0.2463 (12)	0.8804 (18)	0.157 (9)	0.3333
H10G	0.3675	−0.2477	0.9477	0.235*	0.3333
H10H	0.3593	−0.2604	0.8024	0.235*	0.3333
H10I	0.3202	−0.3117	0.8931	0.235*	0.3333
C11C	0.2877 (7)	−0.059 (2)	0.9786 (17)	0.147 (11)	0.3333
H11G	0.3085	−0.0297	1.0463	0.221*	0.3333
H11H	0.2658	−0.1216	1.0089	0.221*	0.3333
H11I	0.2695	0.0114	0.9446	0.221*	0.3333
C12	0.38024 (7)	0.33903 (17)	0.86191 (16)	0.0571 (5)	
C13	0.33690 (10)	0.3607 (2)	0.9473 (2)	0.0868 (7)	
H13A	0.3415	0.3123	1.0227	0.130*	
H13B	0.3076	0.3342	0.9044	0.130*	
H13C	0.3347	0.4490	0.9677	0.130*	
C14	0.42608 (10)	0.3796 (2)	0.9351 (2)	0.0868 (7)	
H14A	0.4310	0.3253	1.0065	0.130*	
H14B	0.4226	0.4656	0.9627	0.130*	
H14C	0.4534	0.3734	0.8822	0.130*	
C15	0.37179 (11)	0.4224 (2)	0.7459 (2)	0.0912 (8)	
H15A	0.3988	0.4144	0.6919	0.137*	
H15B	0.3686	0.5092	0.7712	0.137*	
H15C	0.3427	0.3959	0.7019	0.137*	
C21	0.5064 (4)	0.3703 (4)	0.2258 (7)	0.091 (3)	0.5
H21A	0.5122	0.3683	0.1377	0.136*	0.5
H21B	0.5364	0.3844	0.2713	0.136*	0.5
H21C	0.4841	0.4376	0.2432	0.136*	0.5
O21	0.4864 (2)	0.2543 (3)	0.2625 (6)	0.104 (2)	0.5
H21	0.477 (2)	0.262 (4)	0.342 (2)	0.156*	0.5

### Atomic displacement parameters (Å^2^)

**Table:**

	*U*^11^	*U*^22^	*U*^33^	*U*^12^	*U*^13^	*U*^23^
O1	0.0807 (10)	0.0662 (9)	0.0644 (8)	0.0144 (7)	0.0363 (7)	0.0108 (7)
N1	0.0468 (8)	0.0587 (10)	0.0493 (8)	0.0036 (7)	0.0213 (6)	0.0004 (7)
C1	0.0410 (9)	0.0605 (12)	0.0442 (9)	−0.0018 (8)	0.0135 (7)	−0.0014 (8)
C2	0.0379 (8)	0.0539 (11)	0.0382 (8)	0.0006 (7)	0.0127 (7)	−0.0006 (7)
C3	0.0477 (10)	0.0449 (10)	0.0469 (9)	−0.0005 (8)	0.0149 (7)	−0.0029 (7)
O3A	0.0763 (14)	0.0671 (14)	0.0695 (13)	−0.0293 (11)	0.0309 (10)	−0.0113 (10)
O3B	0.084 (4)	0.047 (3)	0.097 (4)	−0.007 (2)	0.051 (3)	−0.013 (2)
C4	0.0429 (9)	0.0518 (10)	0.0408 (8)	−0.0034 (8)	0.0136 (7)	0.0009 (7)
C5	0.0452 (9)	0.0534 (11)	0.0412 (8)	0.0006 (8)	0.0167 (7)	−0.0032 (7)
C6	0.0486 (9)	0.0479 (10)	0.0379 (8)	0.0013 (8)	0.0056 (7)	−0.0005 (7)
C7	0.0449 (9)	0.0528 (11)	0.0418 (9)	−0.0083 (8)	0.0108 (7)	0.0025 (7)
C8	0.0592 (11)	0.0594 (12)	0.0506 (10)	−0.0138 (9)	0.0203 (8)	−0.0033 (8)
C9A	0.116 (17)	0.135 (15)	0.087 (9)	−0.100 (14)	0.014 (8)	0.013 (9)
C10A	0.074 (7)	0.074 (7)	0.064 (10)	−0.027 (5)	0.000 (7)	0.042 (6)
C11A	0.086 (8)	0.090 (7)	0.27 (2)	−0.063 (6)	0.104 (11)	−0.106 (10)
C9B	0.113 (13)	0.22 (2)	0.203 (18)	−0.129 (15)	−0.082 (12)	0.118 (16)
C10B	0.102 (11)	0.201 (17)	0.068 (10)	−0.064 (10)	0.000 (7)	0.060 (10)
C11B	0.162 (16)	0.141 (11)	0.084 (7)	−0.109 (10)	0.058 (8)	0.002 (8)
C9C	0.076 (10)	0.123 (12)	0.035 (4)	−0.028 (7)	−0.006 (5)	0.011 (5)
C10C	0.198 (17)	0.060 (6)	0.22 (2)	−0.004 (8)	0.148 (16)	0.031 (10)
C11C	0.141 (16)	0.188 (16)	0.120 (11)	−0.147 (12)	0.116 (12)	−0.121 (12)
C12	0.0720 (13)	0.0487 (11)	0.0509 (10)	−0.0004 (9)	0.0072 (9)	−0.0030 (8)
C13	0.1055 (19)	0.0671 (15)	0.0893 (16)	0.0140 (13)	0.0284 (14)	−0.0191 (12)
C14	0.1032 (19)	0.0709 (15)	0.0854 (16)	−0.0121 (13)	−0.0117 (14)	−0.0195 (12)
C15	0.138 (2)	0.0588 (14)	0.0761 (15)	0.0071 (14)	−0.0024 (15)	0.0096 (11)
C21	0.117 (6)	0.079 (3)	0.075 (6)	−0.036 (4)	−0.003 (5)	−0.007 (3)
O21	0.171 (7)	0.0712 (19)	0.072 (3)	−0.031 (3)	0.037 (3)	−0.002 (2)

### Geometric parameters (Å, º)

**Table:**

O1—C1	1.228 (2)	C11A—H11C	0.9600
N1—C1	1.328 (2)	C9B—H9BA	0.9600
N1—C2	1.433 (2)	C9B—H9BB	0.9600
N1—H1	0.95 (2)	C9B—H9BC	0.9600
C1—C1^i^	1.524 (3)	C10B—C11B	1.57 (2)
C2—C7	1.386 (2)	C10B—H10D	0.9600
C2—C3	1.386 (2)	C10B—H10E	0.9600
C3—C4	1.390 (2)	C10B—H10F	0.9600
C3—O3B	1.434 (5)	C11B—H11D	0.9600
C3—H3A	0.9300	C11B—H11E	0.9600
O3A—C7	1.379 (2)	C11B—H11F	0.9600
O3A—H3BA	0.878 (19)	C9C—H9CA	0.9600
O3B—H3BB	0.85 (2)	C9C—H9CB	0.9600
C4—C5	1.387 (2)	C9C—H9CC	0.9600
C4—C8	1.539 (2)	C10C—H10G	0.9600
C5—C6	1.384 (2)	C10C—H10H	0.9600
C5—H5A	0.9300	C10C—H10I	0.9600
C6—C7	1.407 (2)	C11C—H11G	0.9600
C6—C12	1.534 (2)	C11C—H11H	0.9600
C7—H7A	0.9300	C11C—H11I	0.9600
C8—C9B	1.479 (12)	C12—C14	1.529 (3)
C8—C9C	1.495 (10)	C12—C15	1.533 (3)
C8—C10B	1.483 (12)	C12—C13	1.543 (3)
C8—C9A	1.495 (12)	C13—H13A	0.9600
C8—C11B	1.515 (11)	C13—H13B	0.9600
C8—C11C	1.522 (10)	C13—H13C	0.9600
C8—C10A	1.530 (10)	C14—H14A	0.9600
C8—C11A	1.530 (10)	C14—H14B	0.9600
C8—C10C	1.542 (11)	C14—H14C	0.9600
C9A—H9AA	0.9600	C15—H15A	0.9600
C9A—H9AB	0.9600	C15—H15B	0.9600
C9A—H9AC	0.9600	C15—H15C	0.9600
C10A—H10A	0.9600	C21—O21	1.404 (5)
C10A—H10B	0.9600	C21—H21A	0.9600
C10A—H10C	0.9600	C21—H21B	0.9600
C11A—H11A	0.9600	C21—H21C	0.9600
C11A—H11B	0.9600	O21—H21	0.900 (10)
			
C1—N1—C2	129.16 (17)	H9BB—C9B—H9BC	109.5
C1—N1—H1	113.3 (11)	C8—C10B—C11B	59.4 (8)
C2—N1—H1	117.2 (11)	C8—C10B—H10D	109.5
O1—C1—N1	125.80 (16)	C11B—C10B—H10D	108.6
O1—C1—C1^i^	121.01 (19)	C8—C10B—H10E	109.5
N1—C1—C1^i^	113.2 (2)	C11B—C10B—H10E	141.8
C7—C2—C3	120.83 (14)	H10D—C10B—H10E	109.5
C7—C2—N1	123.10 (15)	C8—C10B—H10F	109.5
C3—C2—N1	116.03 (15)	C11B—C10B—H10F	53.4
C2—C3—C4	121.21 (15)	H10D—C10B—H10F	109.5
C2—C3—O3B	120.8 (2)	H10E—C10B—H10F	109.5
C4—C3—O3B	118.0 (2)	C8—C11B—C10B	57.4 (7)
C2—C3—H3A	119.4	C8—C11B—H11D	109.5
C4—C3—H3A	119.4	C10B—C11B—H11D	166.6
C7—O3A—H3BA	104 (3)	C8—C11B—H11E	109.5
C3—O3B—H3BB	152 (6)	C10B—C11B—H11E	75.0
C5—C4—C3	116.60 (15)	H11D—C11B—H11E	109.5
C5—C4—C8	121.79 (14)	C8—C11B—H11F	109.5
C3—C4—C8	121.60 (15)	C10B—C11B—H11F	79.9
C6—C5—C4	124.31 (15)	H11D—C11B—H11F	109.5
C6—C5—H5A	117.8	H11E—C11B—H11F	109.5
C4—C5—H5A	117.8	C8—C9C—H9CA	109.5
C5—C6—C7	117.44 (15)	C8—C9C—H9CB	109.5
C5—C6—C12	122.08 (15)	H9CA—C9C—H9CB	109.5
C7—C6—C12	120.48 (15)	C8—C9C—H9CC	109.5
O3A—C7—C2	123.86 (16)	H9CA—C9C—H9CC	109.5
O3A—C7—C6	116.48 (17)	H9CB—C9C—H9CC	109.5
C2—C7—C6	119.62 (15)	C8—C10C—H10G	109.5
C2—C7—H7A	120.2	C8—C10C—H10H	109.5
C6—C7—H7A	120.2	H10G—C10C—H10H	109.5
C9B—C8—C10B	138.9 (10)	C8—C10C—H10I	109.5
C9B—C8—C11B	114.2 (13)	H10G—C10C—H10I	109.5
C10B—C8—C11B	63.1 (10)	H10H—C10C—H10I	109.5
C9C—C8—C11C	109.5 (11)	C8—C11C—H11G	109.5
C9A—C8—C10A	93.0 (12)	C8—C11C—H11H	109.5
C9B—C8—C4	105.8 (7)	H11G—C11C—H11H	109.5
C9C—C8—C4	114.2 (7)	C8—C11C—H11I	109.5
C10B—C8—C4	114.1 (8)	H11G—C11C—H11I	109.5
C9A—C8—C4	110.1 (8)	H11H—C11C—H11I	109.5
C11B—C8—C4	107.2 (6)	C14—C12—C15	110.89 (19)
C11C—C8—C4	110.6 (7)	C14—C12—C6	109.83 (17)
C10A—C8—C4	108.9 (6)	C15—C12—C6	109.87 (16)
C9A—C8—C11A	102.5 (12)	C14—C12—C13	107.51 (18)
C10A—C8—C11A	124.5 (10)	C15—C12—C13	106.92 (19)
C4—C8—C11A	114.5 (6)	C6—C12—C13	111.78 (16)
C9C—C8—C10C	86.6 (12)	C12—C13—H13A	109.5
C11C—C8—C10C	126.9 (11)	C12—C13—H13B	109.5
C4—C8—C10C	107.1 (6)	H13A—C13—H13B	109.5
C8—C9A—H9AA	109.5	C12—C13—H13C	109.5
C8—C9A—H9AB	109.5	H13A—C13—H13C	109.5
H9AA—C9A—H9AB	109.5	H13B—C13—H13C	109.5
C8—C9A—H9AC	109.5	C12—C14—H14A	109.5
H9AA—C9A—H9AC	109.5	C12—C14—H14B	109.5
H9AB—C9A—H9AC	109.5	H14A—C14—H14B	109.5
C8—C10A—H10A	109.5	C12—C14—H14C	109.5
C8—C10A—H10B	109.5	H14A—C14—H14C	109.5
H10A—C10A—H10B	109.5	H14B—C14—H14C	109.5
C8—C10A—H10C	109.5	C12—C15—H15A	109.5
H10A—C10A—H10C	109.5	C12—C15—H15B	109.5
H10B—C10A—H10C	109.5	H15A—C15—H15B	109.5
C8—C11A—H11A	109.5	C12—C15—H15C	109.5
C8—C11A—H11B	109.5	H15A—C15—H15C	109.5
H11A—C11A—H11B	109.5	H15B—C15—H15C	109.5
C8—C11A—H11C	109.5	O21—C21—H21A	109.5
H11A—C11A—H11C	109.5	O21—C21—H21B	109.5
H11B—C11A—H11C	109.5	H21A—C21—H21B	109.5
C8—C9B—H9BA	109.5	O21—C21—H21C	109.5
C8—C9B—H9BB	109.5	H21A—C21—H21C	109.5
H9BA—C9B—H9BB	109.5	H21B—C21—H21C	109.5
C8—C9B—H9BC	109.5	C21—O21—H21	108.0 (19)
H9BA—C9B—H9BC	109.5		
			
C2—N1—C1—O1	3.7 (3)	C5—C4—C8—C9C	113.4 (10)
C2—N1—C1—C1^i^	−176.68 (17)	C3—C4—C8—C9C	−65.2 (10)
C1—N1—C2—C7	−36.0 (3)	C5—C4—C8—C10B	−102.4 (9)
C1—N1—C2—C3	146.33 (18)	C3—C4—C8—C10B	79.0 (9)
C7—C2—C3—C4	0.5 (3)	C5—C4—C8—C9A	132.7 (12)
N1—C2—C3—C4	178.20 (15)	C3—C4—C8—C9A	−45.9 (12)
C7—C2—C3—O3B	−176.8 (3)	C5—C4—C8—C11B	−34.7 (11)
N1—C2—C3—O3B	1.0 (4)	C3—C4—C8—C11B	146.7 (11)
C2—C3—C4—C5	−0.5 (2)	C5—C4—C8—C11C	−10.7 (11)
O3B—C3—C4—C5	176.8 (3)	C3—C4—C8—C11C	170.7 (11)
C2—C3—C4—C8	178.18 (16)	C5—C4—C8—C10A	−126.6 (6)
O3B—C3—C4—C8	−4.5 (4)	C3—C4—C8—C10A	54.8 (7)
C3—C4—C5—C6	0.4 (3)	C5—C4—C8—C11A	17.8 (10)
C8—C4—C5—C6	−178.29 (16)	C3—C4—C8—C11A	−160.8 (10)
C4—C5—C6—C7	−0.2 (3)	C5—C4—C8—C10C	−152.5 (9)
C4—C5—C6—C12	179.59 (16)	C3—C4—C8—C10C	28.9 (9)
C3—C2—C7—O3A	177.44 (18)	C9B—C8—C10B—C11B	−96.9 (19)
N1—C2—C7—O3A	−0.1 (3)	C4—C8—C10B—C11B	97.8 (8)
C3—C2—C7—C6	−0.3 (2)	C9B—C8—C11B—C10B	134.3 (12)
N1—C2—C7—C6	−177.86 (15)	C4—C8—C11B—C10B	−108.8 (10)
C5—C6—C7—O3A	−177.73 (16)	C5—C6—C12—C14	117.7 (2)
C12—C6—C7—O3A	2.5 (2)	C7—C6—C12—C14	−62.5 (2)
C5—C6—C7—C2	0.2 (2)	C5—C6—C12—C15	−120.0 (2)
C12—C6—C7—C2	−179.65 (15)	C7—C6—C12—C15	59.8 (2)
C5—C4—C8—C9B	87.6 (12)	C5—C6—C12—C13	−1.5 (2)
C3—C4—C8—C9B	−91.0 (12)	C7—C6—C12—C13	178.31 (17)

Symmetry code: (i) −*x*+1, −*y*, −*z*+1.

### Hydrogen-bond geometry (Å, º)

**Table:**

*D*—H···*A*	*D*—H	H···*A*	*D*···*A*	*D*—H···*A*
O3*A*—H3*BA*···O1	0.88 (2)	1.71 (2)	2.578 (2)	167 (4)
O3*B*—H3*BB*···O21^ii^	0.85 (2)	2.14 (2)	2.612 (9)	114 (4)
N1—H1···O21^i^	0.95 (2)	2.27 (2)	3.140 (6)	152 (2)
O21—H21···O1	0.90 (1)	2.01 (3)	2.744 (6)	138 (4)
N1—H1···O1^i^	0.95 (2)	2.20 (2)	2.685 (2)	110 (1)
N1—H1···O3*B*	0.95 (2)	2.41 (2)	2.749 (5)	100 (1)

Symmetry codes: (i) −*x*+1, −*y*, −*z*+1; (ii) *x*, −*y*, *z*+1/2.
